# Identification of cellular factors associated with inflammation and neurodegeneration in multiple sclerosis

**DOI:** 10.3389/fimmu.2025.1648725

**Published:** 2025-08-07

**Authors:** Alexander Rodero-Romero, José Ignacio Fernández-Velasco, Enric Monreal, Raquel Sainz-Amo, Roberto Álvarez-Lafuente, Manuel Comabella, Lluís Ramió-Torrentà, José M. García-Domínguez, Noelia Villarrubia, Susana Sainz de la Maza, María Domínguez-Mozo, Ana Quiroga-Varela, Juan Luís Chico-García, Fernando Rodriguez-Jorge, José Luis Veiga-Gonzalez, Ernesto Roldán-Santiago, Mercedes Espiño, Eulalia Rodríguez-Martín, Gary Álvarez, Jaime Masjuan, Xavier Montalban, Lucienne Costa-Frossard, Luisa María Villar

**Affiliations:** ^1^ Department of Immunology, Hospital Universitario Ramón y Cajal, Red Española de Esclerosis Múltiple (REEM), Red de Enfermedades Inflamatorias (REI), ISCIII, Instituto Ramón y Cajal de Investigación Sanitaria, Madrid, Spain; ^2^ Department of Neurology, Hospital Universitario Ramón y Cajal, Red Española de Esclerosis Múltiple (REEM), Red de Enfermedades Inflamatorias (REI), ISCIII, Instituto Ramón y Cajal de Investigación Sanitaria, Madrid, Spain; ^3^ Grupo Investigación de Factores Ambientales en Enfermedades Degenerativas, Instituto de Investigación Sanitaria del Hospital Clínico San Carlos, Madrid, Spain; ^4^ Servei de Neurologia, Centre d’Esclerosi Múltiple de Catalunya, Institut de Recerca Vall d’Hebron, Hospital Universitari Vall d’Hebron, Universitat Autònoma de Barcelona, Barcelona, Spain; ^5^ Center for Networked Biomedical Research on Neurodegenerative Diseases (CIBERNED) - ISCIII, Madrid, Spain; ^6^ Neurodegeneration and Neuroinflammation Research Group, Girona Biomedical Research Institute (IDIBGI-CERCA), Salt, Spain; ^7^ Red de Enfermedades inflamatorias (RD24/0007/0005), Instituto de Salud Carlos III, Madrid, Spain; ^8^ Medical Sciences Department, Faculty of Medicine, Universitat de Girona, Girona, Spain; ^9^ Department of Neurology, Hospital General Universitario Gregorio Marañón, Madrid, Spain

**Keywords:** multiple sclerosis, serum biomarkers, cellular phenotype and function, neurofilament light chain, glial fibrillary acidic protein, demyelinating disease of central nervous system

## Abstract

**Background:**

Serum biomarkers as neurofilament light chain (sNfL) and glial fibrillary acidic protein (sGFAP) enabled early identification of multiple sclerosis (MS) patients at risk of relapse-associated worsening (RAW) or progression independent of relapses (PIRA). However, the immunological mechanisms underlying these clinical phenotypes remain unclear.

**Methods:**

We conducted a cross-sectional study including 117 MS patients and 84 healthy controls (HC). Patients were stratified as NLGL (low sNfL and sGFAP), NH (high sNfL at different levels of sGFAP), and NLGH (low sNfL and high sGFAP). Percentages of blood and cerebrospinal fluid (CSF) mononuclear cells, and intracellular production of cytokines by T and B cells after “*in vitro*” stimulation were analyzed by flow cytometry.

**Results:**

We identified a common inflammatory profile present in the blood of all MS groups comprising significant increases of effector CD4^+^ and CD8^+^ T cells, of memory and antigen-presenting B cells, of CD4^+^ and CD8^+^ T cells producing interferon-gamma, interleukin-17 and tumor necrosis factor-alpha (TNF-α) and of B cells producing TNF-α. Additionally, the highly inflammatory NH group showed lower frequencies of different regulatory subsets (transitional B cells, PDL1^+^ monocytes and Treg cells) compared to HC and increased percentages of CD4^+^ and CD8^+^ T cells producing granulocyte-macrophage colony-stimulating factor and of effector CD56^dim^ NK cells. They also showed lower percentages of Treg in blood and CSF compared to the low inflammatory NLGL group, which also displayed higher frequencies of regulatory CD56^dim^, NKG2A^+^ cells.

**Conclusion:**

All MS patients share increased inflammatory B and T cells, but differ in regulatory or NK subsets, which identify highly inflammatory or benign disease courses.

## 1 Introduction

MS is the most frequent demyelinating disease of the central nervous system (CNS). It causes inflammation and axonal damage, which are the main causes of disability in MS patients (1, 2).

It is an autoimmune disease caused by a combination of environmental and genetic factors. The immune mechanisms involved in MS have not yet been fully elucidated (1, 3, 4). Multiple studies showed that the disease initiates with peripheral lymphocytic activation in the absence of sufficient regulatory activity (5–7). Stimulated lymphocytes migrate to the CNS, where they are reactivated **by** microglia and resident macrophages. In turn, these lymphocytes further activate these innate immune cells and astrocytes (7–9). Innate and adaptive immune cells release cytokines, chemokines, and other inflammatory mediators, which promote the recruitment of additional immune cells into the CNS. This perpetuates the abnormal immune response that drives inflammation and tissue damage (8, 9).

The course of MS is heterogeneous, ranging from a relatively benign disease to a rapid disability worsening. Identifying patients at risk of having an aggressive disease course is crucial to establish an early-personalized treatment (10). Different immune cell mechanisms have been associated with disease severity. The accumulation of T- and B-cells in the meninges correlates with a worse disease course, indicating the association of intrathecal T- and B-cells with an active disease (11).

Biomarkers in both cerebrospinal fluid (CSF) and serum have been also associated with disease severity and prognosis in MS. These include markers of intrathecal inflammation such as IgM oligoclonal bands (12, 13), activation of the classical and alternative complement pathways (14, 15), chitinase 3-like 1 (CHI3L1), and chemokine ligand 13 (CXCL13) (16, 17), which have been primarily studied in the CSF but can also be detected in serum. Similarly, neurofilament light chains (NfL), components of the axonal cytoskeleton, and glial fibrillary acidic protein (GFAP), a marker of astrogliosis, are detectable in both CSF and serum. When measured in serum, sNfL and sGFAP, offer a less invasive and more accessible alternative to CSF analysis, contributing to identify patients at risk of relapse-associated worsening (RAW) and progression independent of relapse activity (PIRA), respectively (10, 17–24).

The combined assessment of these biomarkers helps to identify distinct disease phenotypes (10, 22, 23). Patients with low levels of both sNfL and sGFAP tend to have a relatively benign disease and respond well to low-, medium-, and high-efficacy treatments. In contrast, patients with elevated sNfL levels, regardless of their sGFAP values, have a worse prognosis, as elevated sNfL has been associated with both RAW, and inflammatory-mediated PIRA (24). These patients typically respond best to high-efficacy therapies, underscoring the importance of systematic monitoring. Finally, patients with low sNfL but high sGFAP levels are at significant risk for non-inflammatory PIRA, which is likely driven by astroglial mechanisms, and these patients tend to have poor responses to current therapies (20, 24).

Although these phenotypes have been well defined, the underlying immunological mechanisms necessary to understand the pathophysiology of the disease and achieve personalized treatments remain unknown. Our aim was to characterize these mechanisms by studying leukocyte subpopulations and cytokine profiles in different clinical groups.

## 2 Methods

### 2.1 Patients and controls

This was a multicenter cross-sectional study including 117 patients with relapsing remitting MS (RRMS) and 84 healthy controls (HC). The Ethics Committee of the Ramon y Cajal University Hospital approved it. Patients and HC were recruited in four university hospitals and all of them provided written informed consent.

The inclusion criteria for MS patients were: to fulfill the 2017 McDonald diagnostic MS criteria (25); age between 18 and 55 years; not having any comorbidities; not being under treatment with any disease-modifying therapy (DMT); having a disease duration of less than a year; and not having received corticosteroids within two months prior to sample collection.

The inclusion criteria for HC were: age between 18 and 55 years; lack of evidence of any disease; absence of toxic habits (alcoholism or drug consumption); and not being under treatment with immunosuppressive drugs or chemotherapeutic agents.

A physician interviewed each candidate before entering in the HC group, although they did not conduct in-depth medical examinations. Participants in the HC group were chosen to be sex- and age-matched compared to MS patients.

We defined a relapse as the emergence of a new neurological symptom or the worsening of an existing symptom, persisting for at least 24 h, in the absence of infection or fever. MS patients were classified as in a relapse if the sample was obtained within 1 month of the attack. Samples collected between 1 and 2 months after a relapse were excluded only when studying differences between patients in a relapse or in remission. No samples were excluded in the remaining analyses performed in this study.

### 2.2 Samples

Sixteen milliliters of peripheral blood were collected from each study participant using two sodium heparin tubes (Sarstedt AG & Co., Nümbrecht, Germany) along with an additional 8 millilitres in a separate dry tube (Sarstedt). Peripheral blood mononuclear cells (PBMCs) were isolated from the heparinised blood using Ficoll/Hypaque (Abbott Laboratories St. Louis, MO, United States) density gradient centrifugation and cryopreserved until analysis in fetal bovine serum (Sigma–Aldrich, Chicago, United States) supplemented with 20% dimethyl-sulfoxide (Sigma–Aldrich). Serum was aliquoted, and stored at −80°C until analysis. Moreover, a lumbar puncture was performed on 82 patients to collect CSF samples.

### 2.3 Monoclonal antibodies

The monoclonal antibodies used in the analysis of PBMCs in this study included CD4 FITC, CCR7 PE, CD3 PE-Cy5-5, CD45RO APC, CD8 APC-Cy7, PD1 BV421, CD45 V500, CD24 FITC, CD27 PE, CD38 PeCy5-5, CD19 PE-Cy7, IgM APC, CD86 APC-Cy7, CD80 Pacific Blue, CD14 FITC, CD25 PE, CD16 PeCy7, CD56 APC, CD127 Pacific Blue-A, CD57 PE, PD-L1 PE-Cy7, CD3 BV421, IFN-γ FITC, GM-CSF PE, TNF-α PerCP-Cy5.5, CD122 PE, NKG2D APC, NKG2C BV421, CD158a FITC, CD158b PE, CD158e APC, NKG2A BV421 (BD Biosciences, San Jose, CA, USA), CXCR5-PE-Cy7 (BioLegend, San Diego, CA, USA) and IL-17 APC (R&D Systems, Minneapolis, MN, USA).

The monoclonal antibodies used in the analysis of CSF cells included CD3-PerCP, CD8-APC-H7, CD27-FITC, CD19-PE-Cy7, CD25-PE, CD14-APC, CD45-V500 and CD127-BV421 (BD Biosciences).

### 2.4 Labelling of surface molecules

The cryopreserved PBMCs were thawed, and viability was evaluated in a Neubauer chamber by the Trypan blue dye exclusion test to assess cell viability (Merck, Darmstadt, Germany). A minimum of 2 x 10^5^ viable cells per tube were labelled with adequate amounts of fluorescence-labelled monoclonal antibodies for 30 min at 4°C in the dark. The cells were washed twice with phosphate-buffered saline (PBS) and analyzed by flow cytometry as described below.

Fresh CSF samples were centrifuged at 500 × g for 10 min. Cellular pellets were resuspended in their residual volume (approximately 100 µl), stained with the appropriate amounts of monoclonal antibodies for 30 min at 4°C in the dark, washed twice with PBS and analyzed by flow cytometry as detailed below.

### 2.5 *In vitro* stimulation and intracellular cytokine staining of PBMCs

We studied the intracellular production of cytokines such as tumour necrosis factor-alpha (TNF-α), interleukin 17 (IL-17), interferon-gamma (IFN-γ) and granulocyte macrophage colony-stimulating factor (GM-CSF).

Aliquots containing 5 x 10^6^ PBMCs were suspended in 1 ml of complete medium (2 mM L-glutamine and 10% fetal bovine serum (Sigma–Aldrich)) supplemented with 50 ng/ml phorbol 12-myristate 13-acetate (PMA) (Sigma–Aldrich) and 750 ng/ml ionomycin (Sigma–Aldrich). Additionally, 2 µg/ml brefeldin A (GolgiPlug, BD Biosciences) and 2.1 µM Monesin (GolgiStop, BD Biosciences) were added. The suspension was incubated in polypropylene tubes at 37°C in a 5% CO2 atmosphere for 4 h.

Following the incubation period, the PBMCs were washed with phosphate-buffered saline (PBS), resuspended and stained in the dark for 30 min at 4°C with appropriate amounts of monoclonal antibodies targeting surface antigens. The cells were subsequently washed with PBS, fixed, and permeabilized for 20 min at 4°C in the dark using a Cytofix/Cytoperm Kit (BD Biosciences). After two washes with Perm/Wash solution (BD Biosciences), the cells were stained intracellularly for 30 min at 4°C in the dark with monoclonal antibodies specific to the following cytokines: IFN-γ, GM-CSF, TNF-α, and IL-17. Two additional washes were subsequently performed, and the PBMCs were analyzed by flow cytometry. Nonstimulated PBMCs were used as controls for basal production.

### 2.6 Flow cytometry

A minimum of 1 x 10^5^ events in PBMC and at least 10^2^ events in CSF were analyzed within 1 h after antigen labeling using a FACSCanto II. (BD, Bioscence). Isotype controls were used to set the mean autofluorescence values. The percentages of every subset of total mononuclear cells obtained were analyzed by the FACSDiva software V.8.0 (BD Biosciences, San Jose, CA, USA).

The gating strategies for the PBMC subsets are shown in **Supplementary Figures S1**, **S2**, and those for the CSF cell subsets are shown in **Supplementary Figure S3**.

### 2.7 sNfL and sGFAP quantification

sNfL and sGFAP levels were assessed by a Simoa NF-light™ Advantage Kit and a Simoa™ GFAP Discovery Kit, respectively (Quanterix, Billerica, MA, USA), on an SR-X instrument (Quanterix, Billerica, MA, USA) following the manufacturer’s instructions.

sNfL values were expressed via the Z-score, which corrects raw values according to age and body mass index (26). Z-scores were calculated using the Serum Neurofilament Light Chain Reference App. Patients were classified according to Z-score and sGFAP levels into one of three groups: NLGL, NH, and NLGH. We established a Z-score < 1.5 for sNfL and 140 pg/ml for sGFAP following normal values established for HC as cut-off values (27).

### 2.8 Statistical analysis

Statistical analyses were performed via GraphPad Prism 9.0 (GraphPad Prism Inc., San Diego, CA, USA). All tests were two-tailed, and p < 0.05 was considered significant.

Categorical variables are expressed as numbers and percentages [n(%)] and differences between groups were analyzed using the chi-square test. Continuous variables were tested for normality using the Shapiro-Wilk test. As not all followed a normal distribution, they were presented as median (interquartile range). Group comparisons for continuous variables were performed using the Kruskal-Wallis test with Dunns *post-hoc* analysis to explore inter-group comparisons.

### 2.9 Data availability

Original data will be available to any researcher in the field for three years by request to the corresponding author.

## 3 Results

We classified the 117 MS patients into three groups on the basis of their sNfL and sGFAP levels, using the cut-off values previously established (27).The first group (n=45) included patients with low sNfL (Z score < 1.5) and low sGFAP (< 140 pg/ml) values (NLGL). The second group (n=53) included patients with high sNfL values (Z score ≥ 1.5, NH). The third one (n=19) included patients with low sNfL values (Z score < 1.5) and high sGFAP ones (≥ 140 pg/ml) (NLGH). The demographic and baseline clinical characteristics of the HC and MS patients are summarized in **Table 1**. No differences in sex or age were found. However, we detected differences in disease duration and gadolinium-enhancing lesions. Disease duration was slightly longer in the NLGH group than in the NH group (p=0.01) and there were more gadolinium-enhancing lesions in the NH group than in the NLGL group (p=0.0001).

**Table 1:** Demographic and clinical data of the cohorts included in the study.

Demographic and clinical data	HC (n=84)	NLGL (n=45)	NH (n=53)	NLGH (n=19)
Age (years)	34.5 [26-45]	40 [36-47]	42 [35.5-47]	38.5 [32-44]
Sex female	55, 65%	30, 67%	36, 68%	13, 68%
Disease duration (days)		54.1 [27.7-72.6]	27.6 [5.6-56.7]	67.1 [12.3-123] #*
Patients with relapse		23, 51.1%	25, 47.2%	11, 57.9%
EDSS at baseline		1.5 [1-2]	1.5 [1.5-2]	1.5 [1.5-2]
Gd-enhancing lesions		0 [0-1]	**2 [0-5]**¶****	0 [0-2]
IgG oligoclonal bands		41 (93)	48 (91)	17 (89)
sNfL levels (pg/ml)	6.2 [4.4-9.2]	6.6 [4.4-7.8]	20.1[15-34.6] ʅ****	7.3 [5.9-8.6]
Z-Score	-0.2 [-1.1-0.65]	-0.8 [-1.6-0.1]	2.4 [2.0-3.0] ʅ****	0.5 [-0.6-0.99]
sGFAP levels (pg/ml)	95 [74.6-116.2]	98.5 [81-114]	**158.3 [116.7-259.6]** ¶**** ⱡ ****	**194.4 [153.9-290.1]** ¶**** ⱡ ****
Topography of first relapse				
Optic nerve		15, (33.3)	8, (15)	3, (15.8)
Brainstem		9, (20)	14, (26.4)	5, (26.3)
Spinal cord		16, (35.7)	20, (37.8)	10, (52.6)
Multifocal		2, (4.4)	2, (3.8)	0, (0)
Others		3, (6.6)	9, (17)	1, (5.3)
T2 lesions at baseline				
0		1 (2.2)	1 (1.9)	0 (0)
1-3		8 (17.8)	4 (7.5)	0 (0)
4-9		16 (35.6)	9 (17)	5 (26)
10-50		19 (42.2)	31 (58.5)	12 (63)
<50		1 (2.2)	8 (15.1)	2 (11)

Continuous variables are expressed as medians and 25-75% interquartile ranges (25-75% IQRs). Differences between groups were examined via the Kruskal–Wallis multiple comparisons test, with Dunn’s *post hoc* test. Continuous variables are presented as medians [25-75% IQR]. Significant comparisons are highlighted in bold. EDSS, Expanded Disability Status Scale; Gd, Gadolinium; HC, Healthy Controls; IQR, Interquartile Range; NH, Patients with high sNfL Z-scores; NLGH, Patients with low sNfL Z-scores and high GFAP levels; NLGL, Patients with low sNfL Z-scores and GFAP values. **ⱡ**: significant difference with the HC group; ʅ: significant difference compared with all three groups; ¶: significant difference compared with the NLGL group; *: p<0.05, **: p<0.01, ***: p<0.001, and ****: p<0.0001 according to Dunn’s test. In all cases, significant p values in Dunn’s test implied p values < 0.05 in the associated Kruskal–Wallis test.

### 3.1 Differences in peripheral blood immune cell subsets

We next explored the different immune cell subsets in the three patient groups and in HC. The results of immune cells subsets are shown in **Table 2** and the results of the cytokine-producing blood immune cells in **Supplementary Table S1**.

**Table 2:** Percentages of peripheral mononuclear blood cell subsets.

Cell population	HC (n=84)	NLGL (n=45)	NH (n=53)	NLGH (n=19)
Lymphocytes	85.9 [81.9-88.3]	86.9 [80.7-90.9]	84.6 [80.2-89]	86.9 [80-90.3]
CD4^+^ T cells	35.7 [29.4-40.8]	35.2 [31.1-42.7]	36.3 [28.9-43.5]	35.6 [31.6-41.7]
Naïve	12.3 [10.3-16.2]	13.4 [8.4-15.3]	10.7 [5.2-15.9]	13.8 [8.9-18.7]
Central Memory	9.1 [6.4-11.8]	7.1 [5.0-12.4]	7.5 [4.3-12.8]	7.2 [3.7-12.7]
Terminally differentiated	5.1 [1.3-7.9]	3.9 [0.7-5.8]	2.8 [1.6-5.7]	4.8 [1.6-7.3]
Effector Memory	4.5 [2.5-9.5]	8.4 [4.7-10.9] ⱡ*	12.0 [5.3-17] ⱡ***	9.9 [6.7-14.5]ⱡ*
Regulatory	1.1 [0.8-1.4]	0.7 [0.5-1.4]#*	0.6 [0.4-1.1]ⱡ****	1.0 [0.8-1.6]
CD8^+^ T cells	16.5 [14.7-19.7]	17.4 [14.6-23.3]	19.0 [15.4-25.2]	18.4 [15.7-21.2]
Naïve	8.0 [5.3-9.7]	4.2 [2.6-7.8] ⱡ**	4.5 [2.8-7.6] ⱡ***	5.6 [4-8.3]
Central Memory	0.7 [0.5-1.1]	0.7 [0.4-1.2]	0.7 [0.5-1.3]	0.6 [0.4-1.2]
Terminally differentiated	3.6 [2.2-5.3]	5.4 [4.4-8.9] ⱡ***	4.6 [2.6-8]	4.9 [3.1-8.3]
Effector Memory	4.1 [2.6-6.1]	5.2 [3.3-8.6]	6.6 [4.1-9.2] ⱡ****	7.3 [3.9-10.3] ⱡ*
CD19^+^ cells	6.0 [4.9-8.5]	5.9 [4.6-8.2]	6.4 [4.5-9.2]	6.4 [5.5-8.4]
Naive	3.8 [2.9-5.6]	2.9 [2.5-5.1]	3.4 [2.2-5.2]	3.8 [2.6-5.6]
Memory	1.6 [1.3-2.5]	2.3 [1.8-3.4] ⱡ*	2.4 [1.6-3.4] ⱡ*	3 [1.6-3.6] ⱡ*
Plasmablasts	0.08 [0.05-0.14]	0.06 [0.03-0.1]	0.07 [0.04-0.11]	0.07 [0.04-0.12]
Transitional	0.3 [0.1-0.6]	0.2 [0.07-0.4]	0.1 [0.06-0.3] ⱡ**	0.2 [0.1-0.4]
CD80^+^	0.8 [0.6-1.2]	1.2 [0.7-1.7] ⱡ*	1.2 [0.9-1.7] ⱡ***	1.1 [0.8-1.3]
CD86^+^	1.8 [1.4-2.7]	2.9 [1.6-4.9] ⱡ*	3.5 [1.1-4.8] ⱡ**	3.5 [2.7-5.3] ⱡ*
NK CD56^dim^	11.2 [8.2-14.8]	8.9 [6.6-12.8] #****	14.8 [11.2-19.5] ⱡ**	9.1 [7.1-11.2] #***
NK CD56^dim^ CD122^+^	9.7 [7.1-13]	8.5 [6.9-11.7] #***	12.6 [9.9-18.4] ⱡ*	8.5 [5.8-11.5] #***
NK CD56^bright^	0.8 [0.4-1.1]	0.9 [0.7-1.6]	0.7 [0.4-1.1]	0.7 [0.5-1.2]
NKT	2.3 [1.2-3.6]	2.6 [1.5-3.8]	2.8 [1.9-4.4]	2.7 [1.3-8.5]
Monocytes	12.5 [10.2-16.3]	9.7 [6.7-16.0]	12.7 [6.3-18.5]	12.2 [7.2-17.1]
PD-L1^+^	0.3 [0.1-1.2]	0.2 [0.1-0.5]	0.2 [0.1-0.3] ⱡ*	0.4 [0.1-0.9]

The percentage values are expressed as medians [25-75% IQR]. We calculated all percentages of CD45^+^ cells. Significant comparisons are highlighted in bold. HC, healthy controls; IQR, interquartile range; NH, patients with high sNfL Z-scores; NKs, natural killer cells; NLGH, patients with low sNfL Z-scores and high GFAP levels; NLGL, patients with low sNfL Z-scores and GFAP values; PD-L1, programmed death-Ligand 1. **ⱡ**: significant difference with the HC group; #: significant difference with the NH group; *p<0.05, ** p<0.01, *** p<0.001, and **** p<0.0001 by Dunn’s test. In all cases, significant p values in Dunn’s test implied p values < 0.05 in the associated Kruskal–Wallis test.

All MS groups presented some common differences compared to HC. They showed an increase in the percentages of effector memory CD4^+^ T-cells (the difference was most notable in the NH cohort), memory B-cells, and CD86^+^ antigen-presenting B-cells. MS groups also presented increased percentages of effector CD8^+^ T-cells. Differences were due to effector memory CD8^+^ T-cells in the NH and NLGH groups (p=0.001 for both) and to terminally differentiated CD8^+^ T-cells (p=0.0001) in the NLGL group.

**Figure 1:**
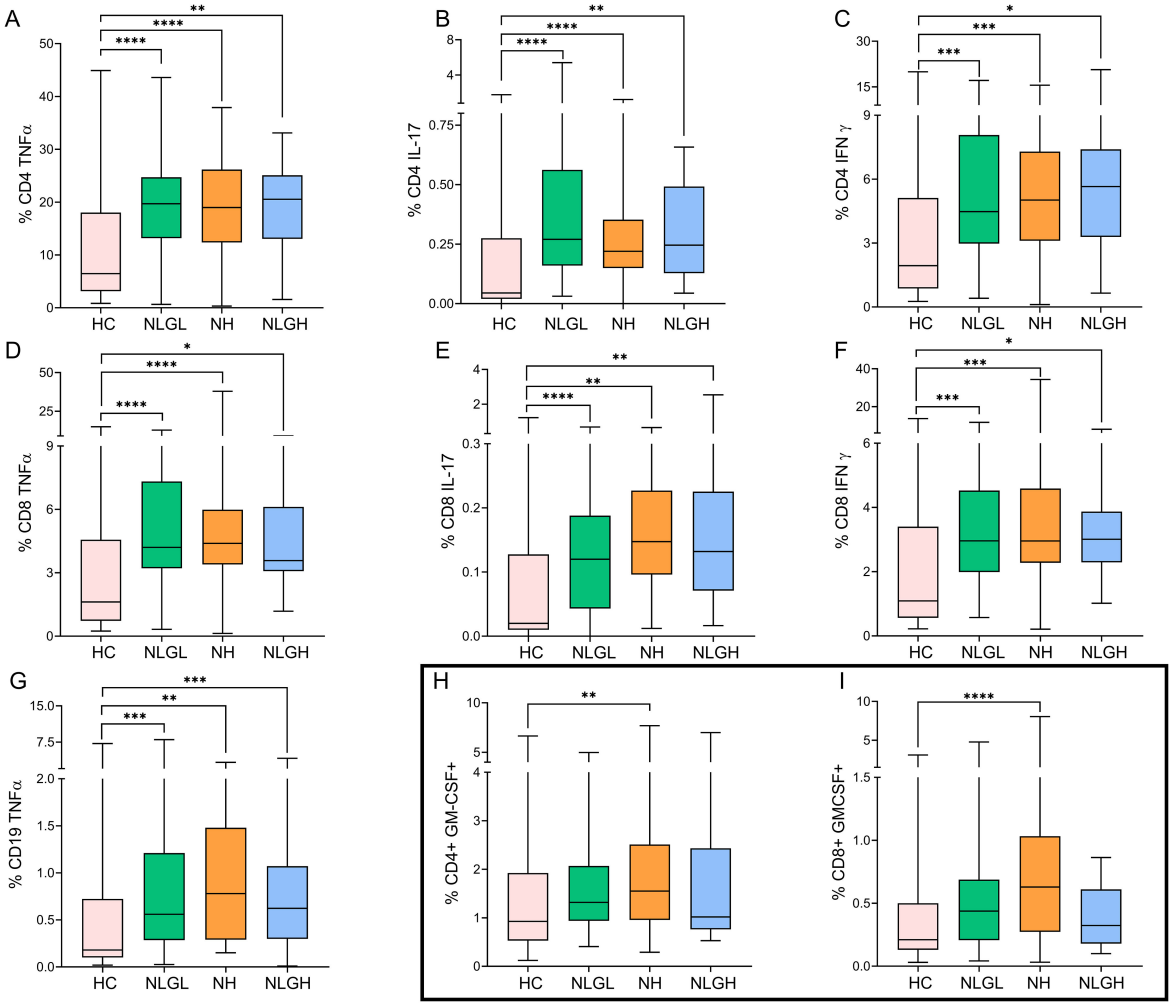
Blood percentages of intracellular cytokine-producing cells in the MS group and HC. Percentages of CD4^+^ T cells producing TNFα **(A)**, IL-17 **(B)**, or IFN-γ **(C)**; CD8^+^ T cells producing TNFα **(D)**, IL-17 **(E)**, or IFN-γ **(F)**; CD19^+^ B cells producing TNFα **(G)**; and CD4^+^ **(H)** and CD8^+^ **(I)** T cells producing GM-CSF. The percentages of the subpopulations are shown on CD45^+^ cells. HC, healthy controls; MS, multiple sclerosis; NH, patients with high sNfL Z-scores; NLGH, patients with low sNfL Z-scores and high GFAP levels; NLGL, patients with low sNfL Z-scores and GFAP values. *p<0.05, **p<0.01, ***p<0.001, and ****p<0.0001 according to Dunn’s multiple comparisons test.

Additionally, all groups presented increased percentages of CD4^+^ and CD8^+^ T-cells that produced TNF-α (**Figures 1A, D**), IL-17 (**Figures 1B, E**), and IFN-γ (**Figures 1C, F**) and increased percentages of CD19^+^ B-cells that produced TNF-α (**Figure 1G**).

On the other hand, the NLGL and NH groups presented specific differences with HC, not observed for the NLGH group. They showed increased percentages of CD80^+^ antigen-presenting B cells (p=0.01 for both groups) and a decrease in the proportion of naïve CD8^+^ T-cells (p=0.001 for both groups).

**Figure 2:**
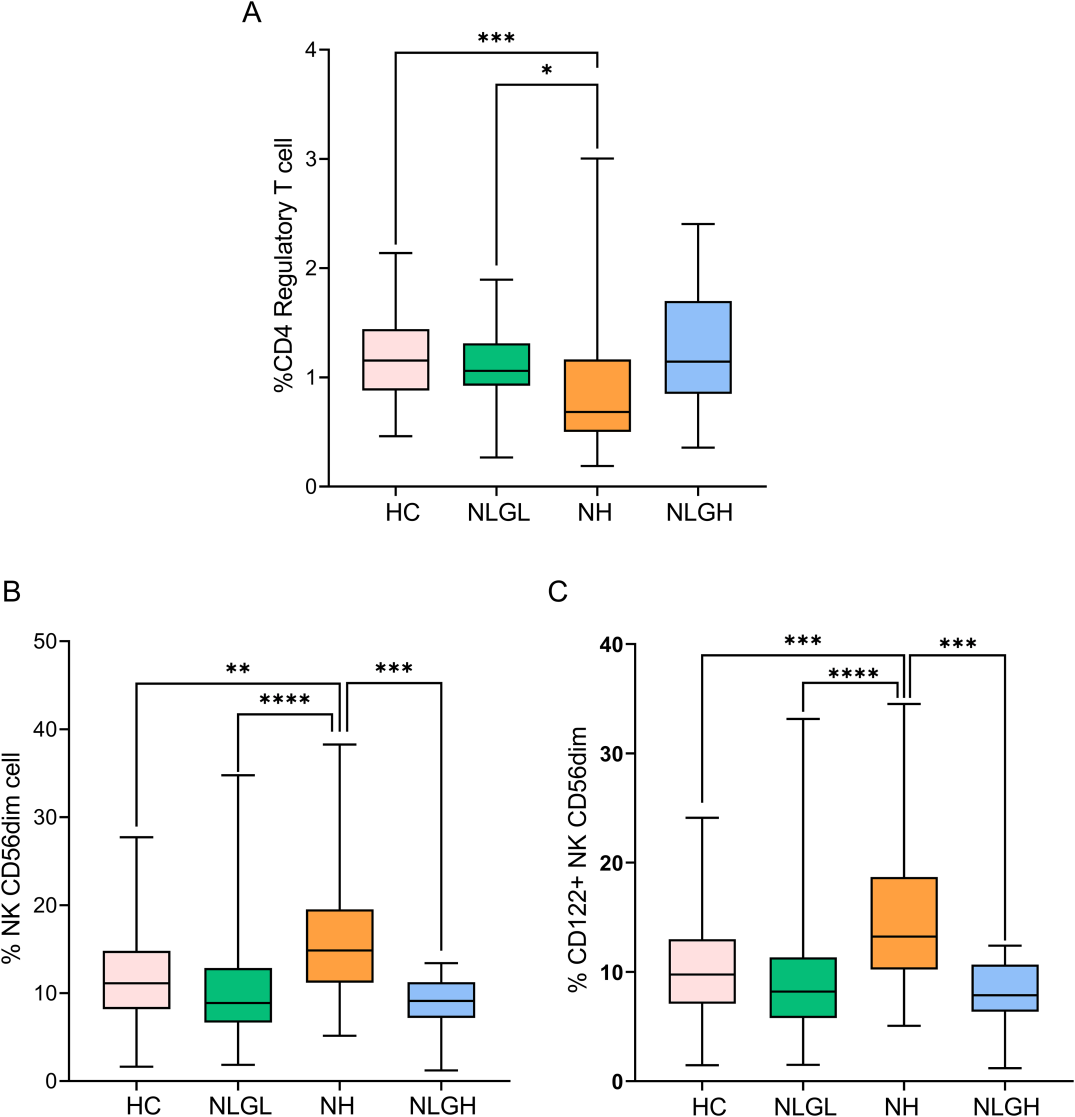
Blood percentages of CD4+ regulatory T-cells **(A)**, NK CD56^dim^ cells **(B)**, and CD122^+^ NK CD56^dim^ cells **(C)**. The percentages of the subpopulations are shown on CD45^+^ cells. HC, healthy controls; NH, patients with high sNfL Z-scores; NLGH, patients with low sNfL Z-scores and high GFAP levels; NLGL, patients with low sNfL Z-scores and GFAP values. *p<0.05, **p<0.01, ***p<0.001, and ****p<0.0001 according to Dunn’s multiple comparisons test.

We also identified unique characteristics for different MS groups. Thus, the NLGL group presented a marked increase in the percentages of regulatory CD56^dim^ NKG2A^+^ NK cells compared with the HC (p=0.0001) and the NH groups (p=0.0001) (**Supplementary Table S2**). By contrast, the NH group displayed notable reductions in the proportions of the following regulatory cells: transitional CD19^+^ B-cells (p=0.002), programmed death-ligand 1^+^ (PD-L1) monocytes (p=0.01), and regulatory CD4^+^ T-cells (Treg) (p=0.0001, **Figure 2A**) compared with HC, being the differences in Treg also observed when compared NH with NLGL patients (p=0.017).

NH patients also presented increased percentages of CD4^+^ (p=0.004, **Figure 1H**) and CD8^+^ (p<0.0001, **Figure 1I**) T-cells that produce GM-CSF.

The most clear differences observed in NH group were the increase in the proportion of CD56^dim^ NK cells compared to HC (p=0.002), NLGL (p=0.001) and NLGH (p=0.0002) groups (**Figure 2B**). This was mainly due to CD56 ^dim^ cells expressing CD122^+^ (p=0.0004, compared to HC, p<0.0001 compared to NLGL and p=0.0001 compared to NLGH, **Figure 2C**).

We further explored if sGFAP could identify some differences in NH group and studied the differences in blood immune cell subsets in NH patients with low (NHGL, n=19) and high (NHGH, n=34) sGFAP values. We did not find any differences between them in any of the immune cells explored in this study (**Supplementary Table S3**).

### 3.2 Differences in the immune cell subsets present in CSF

We also performed CSF immune cell staining in 82 of our patients. Forty-four patients belonged to the NH group, 30 to the NLGL group and 8 to the NLGH group. A protocol was established for the analysis of CD4^+^, CD8^+^, CD14^+^ and CD19^+^ cell subsets. The results are shown in **Supplementary Table S4**.

**Figure 3:**
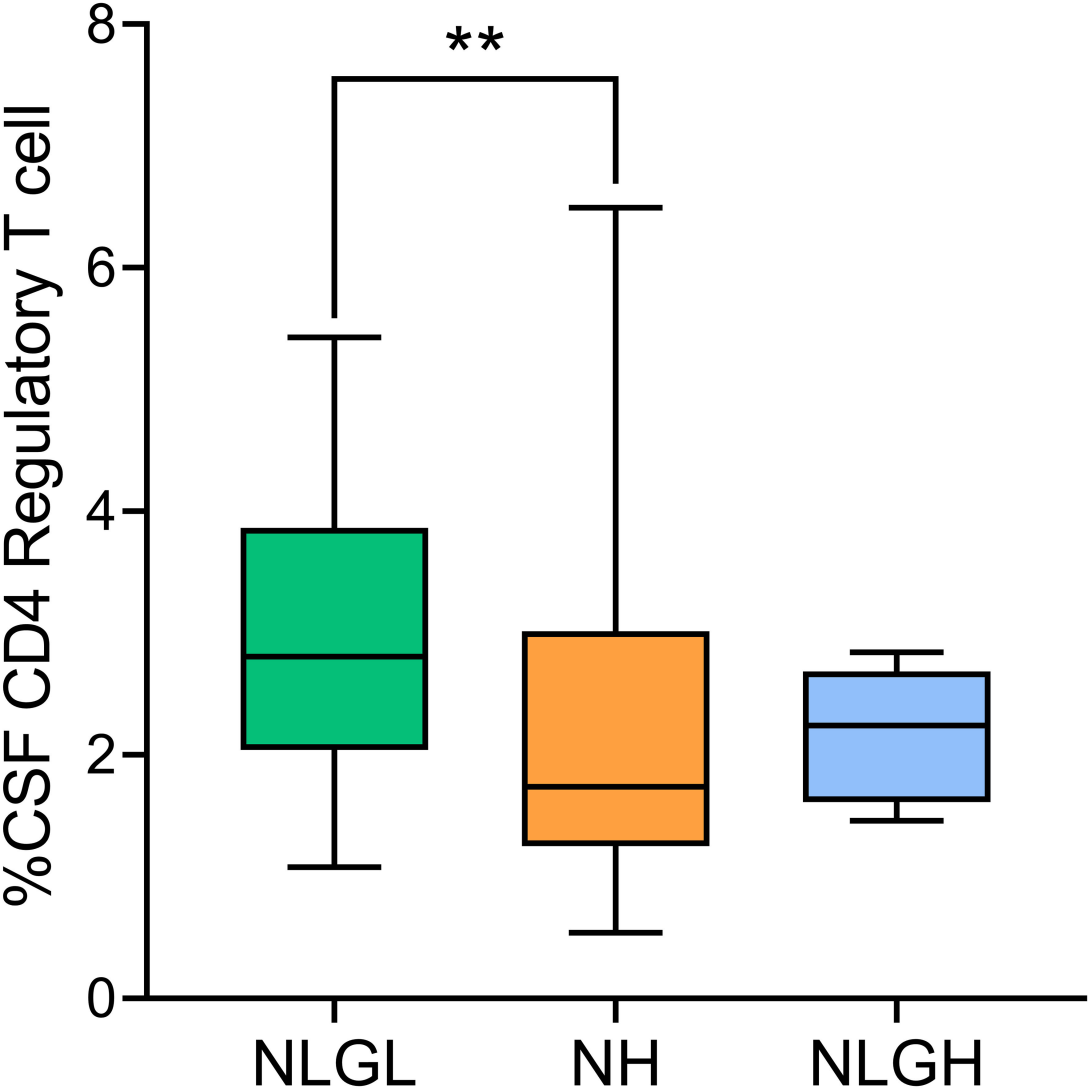
Percentages of regulatory T-cells in the cerebrospinal fluid (CSF) of MS patients. Regulatory T-cells were defined as CD25^high^ CD127^-^ cells. NH, high sNfL Z score group; NLGH, patients with low sNfL Z-scores and high GFAP levels; NLGL, patients with low sNfL Z-scores and low GFAP levels; **p<0.01 according to Dunn’s multiple comparisons test.

The only difference observed between groups was a clear decrease in the percentage of Treg showed by NH patients compared with that in NLGL patients (p=0.0019, **Figure 3**), thus confirming the results obtained in the peripheral blood.

## 4 Discussion

The discovery of serum biomarkers has changed the prognosis and follow-up of MS patients. They helped identify, at disease onset, patients with a relatively benign course, those at risk for RAW and those prone to PIRA (19, 22, 24). However, much less is known about the biological processes underlying these different disease trajectories. Understanding the immunological basis behind MS phenotypes is of utmost clinical relevance, as it may facilitate more accurate treatment decisions and uncover novel therapeutic targets.

To explore this, we analyzed leukocyte subpopulations and cytokine profiles in recently diagnosed MS patients, stratified according to sNfL and sGFAP levels (24) and age- and sex-matched HC for comparison.

We found that all MS subgroups showed increased percentages of effector T cells, memory and antigen-presenting B cells, and CD4^+^ and CD8^+^ T cells producing TNF-α, IL-17, and IFN-γ, as well as CD19^+^ B cells secreting TNF-α compared with HC. Previous studies described the expansion of effector and pro-inflammatory T cells in both blood and CNS tissue of MS patients (28–30). Additionally, MS patients showed increased memory B cells in the CSF, and higher percentages of antigen-presenting B cells in the peripheral blood (31, 32). However, little is known about the association of any of these T and B cell subsets with the different inflammatory or neurodegenerative profiles. Our work shows that the blood upregulation of these activated and memory T and B cell subsets is a common feature of the disease, and even patients with low risk of RAW and high probability of a smoldering disease (24) share this proinflammatory lymphocyte profile. This highly suggests that differences between the different clinical profiles may be associated to other immune cell subsets. In this line, we identified distinct immunological profiles between the MS subgroups. In particular, the NH group showed a clear reduction in the percentages of regulatory T cells (Treg) in peripheral blood compared to HC and in both blood and CSF compared to the NLGL group. Several studies suggested that changes in the number or function of Treg may play a role in MS pathology (33–37). However this data could not be reproduced in different works. Some studies reported no significant differences in Treg frequency in MS patients (33), or even reported that Treg may convert into activated, pro-inflammatory T cells in other neurological diseases (37). Others described functional deficit in the Treg subset (34–37), rather than a simple decrease in Treg numbers. These previous studies analyzed total MS patients. We observed that low Treg values clearly associate with a highly inflammatory disease, demonstrated by elevated sNfL values (NH group). This shows that low Treg numbers is not a common characteristic of all MS patients but of highly inflammatory ones. This reduction was also found in the CSF, suggesting that the lack of Treg in the blood may also affect the immune environment within the CNS. This supports the idea that a decrease in regulatory T cells could lead to poor control of inflammation both in the periphery and in the CNS, contributing to the highly inflammatory profile shown by this group of patients (24).

NH patients also exhibited decreased counts of transitional B cells, known for their regulatory properties and potential to ameliorate MS severity (38), as well as reduced percentages of PD-L1^+^ monocytes, which exert immunomodulatory effects through inhibition of T cell activity (39). The reduction of these additional regulatory subsets may contribute to poor regulation of the inflammatory response, contributing to the highly inflammatory course in the NH group. Supporting this, NH patients also displayed an increased frequency of GM-CSF–producing CD4^+^ and CD8^+^ T cells, which have been implicated in the activation of innate immune cells and correlate with MS disease activity, as high blood and CSF levels associate with worse outcomes (40, 41).

Finally, NH patients also exhibited a marked increase in the percentage of CD56^dim^ NK cells. Although data on this NK cell subset in MS are limited, these cells have been shown to induce perivascular cortical demyelination, both in biopsies from MS patients and in an experimental model of the disease (42). In contrast, CD56^bright^ NK cells are typically associated with regulatory functions, and their expansion has been observed in optimal responders to certain disease-modifying therapies (43). In our study, the majority of CD56^dim^ NK cells expanded in NH patients expressed CD122, the intermediate-affinity IL-2 receptor (44). Given the pronounced reduction of Treg, which express high levels of CD25, the high-affinity IL-2 receptor (33) in NH patients, it is plausible that CD56^dim^ NK cells have increased access to IL-2 in this context, promoting their expansion and cytotoxic potential. These findings further support the notion of a dysregulated immune environment in NH patients, where a loss of regulatory control coincides with the expansion of potentially pathogenic subsets. In this inflammatory milieu, CD56dim NK cells may actively contribute to axonal damage.

To rule out if differences found in the NH group could be dynamic and secondary to the proximity to the relapse or an intrinsic characteristic of the patient’s immune milieu in the long term we explored differences in time from a relapse in our three groups. No differences between NLGH and NH groups were found despite the different immune cell profiles found in these groups. We did not find differences in the proportion of patients being in a relapse in the three groups either. This strongly suggests that differences found in NH group associate with a higher inflammatory disease course independently of the presence of clinical relapses.

We also observed an interesting NK cell profile in the NLGL group. These patients presented increased percentages of CD56^dim^ cells expressing NKG2A, compared to HC and to NH. NKG2A serves as a key inhibitory checkpoint that can limit NK cell–mediated cytotoxicity and proinflammatory responses, thus promoting immune tolerance (45). This may contribute to a better control of inflammatory activity in these patients, who show a relatively benign disease course.

Finally, we did not detect unique peripheral immune characteristics in the NLGH group, apart from the common inflammatory profile observed in all MS groups. The elevated sGFAP levels shown by these patients could indicate a CNS-restricted inflammatory process (46, 47). Indeed, they reflect astrocyte activation, which has been linked to disease progression and to chronic tissue injury, particularly in the setting of smoldering, compartmentalized inflammation within the CNS (22, 24, 44, 47). However, progression in these patients could also be mediated by a non-immune mediated process. Future studies in CSF with a wider panel of immune cell subsets analysed will further elucidate this.

This study has some limitations. The number of patients in the NLGH group was relatively small, especially for CSF analyses. This could prevent us from finding additional differences in this group of patients. In addition, a prospective follow-up was not made to explore the association of any of the cellular biomarkers described in this study with patient outcomes. Moreover, although we did not find differences in the proportion of patients in which samples were obtained during a relapse, the higher number of gadolinium-enhancing lesions in the NH group may indicate that findings observed in this group of patients could associate with increased disease activity at sampling. This could be a potential limitation of the study. Finally, our results were not validated in independent cohorts. Future research will address these issues to further demonstrate the association of the blood leukocyte subsets and patient outcomes.

In summary, our results show common immune mechanisms characteristic of all MS patients and particular subsets associated with highly inflammatory and benign disease courses. These findings demonstrate that stratification by sNfL and sGFAP levels is associated with distinct immunological mechanisms and support the utility of serum biomarkers not only for prognosis but also for biological characterization of MS phenotypes. Validation of these findings in independent multicenter cohorts is needed to further demonstrate their potential for guiding personalized treatment strategies.

## Data Availability

The raw data supporting the conclusions of this article will be made available by the authors, without undue reservation.
