# Supplementary material for: Identification of cellular factors associated with inflammation and neurodegeneration in multiple sclerosis
Source: Front Immunol. 2025 Aug 7;16:1648725. doi: 10.3389/fimmu.2025.1648725 (PMC12367507; doi:10.3389/fimmu.2025.1648725)
Supplement: Supplementary file 1 [file DataSheet1.docx]

Supplementary Material


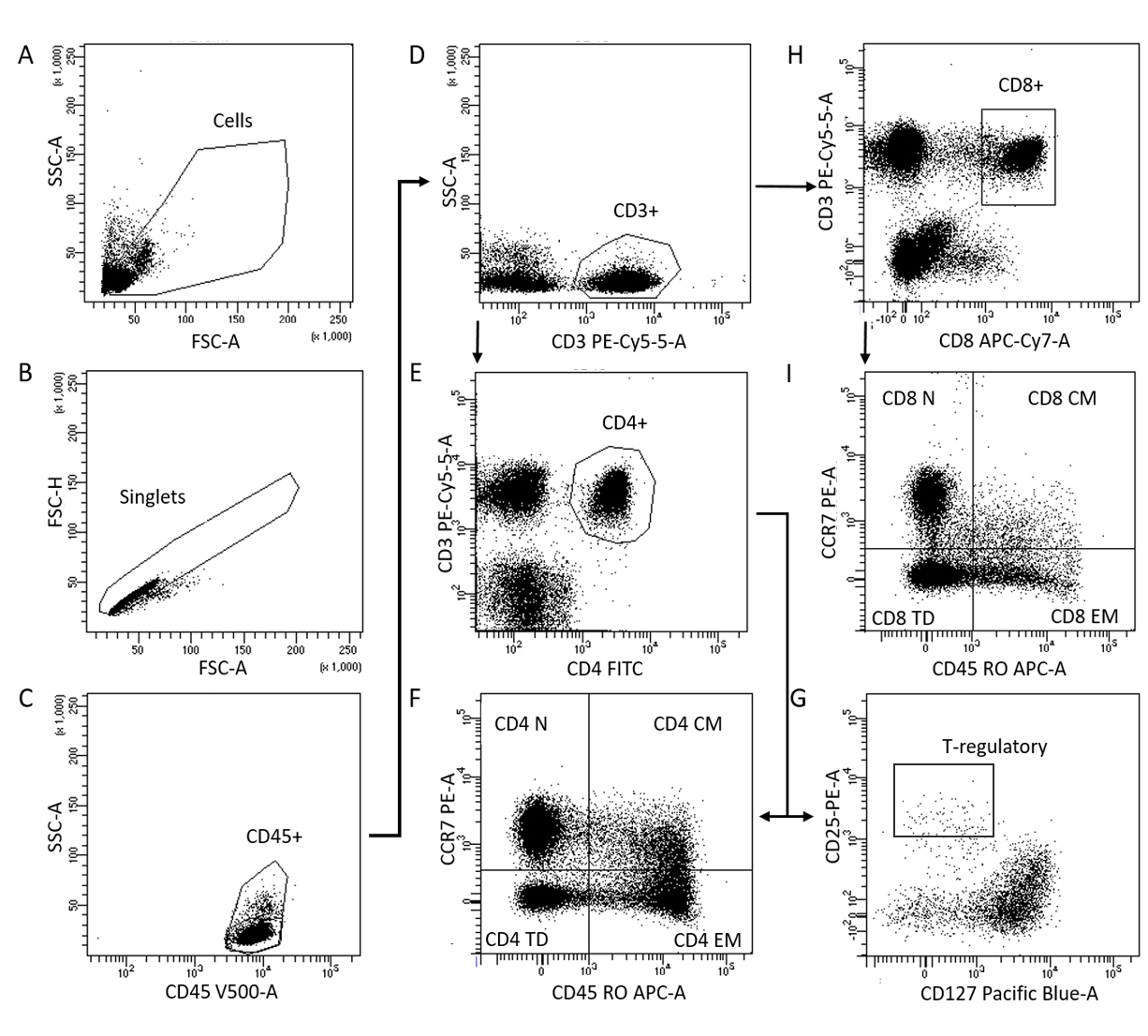


Supplementary Figure 1. Gating strategy for T-cells. Selection of viable cells based on forward scatter (FSC) and side scatter (SSC) characteristics (A). Doublets were excluded from the analysis (B). CD45^+^ cells were gated (C). T-cells were identified as CD45^+^ and CD3^+^ cells (D). T helper cells were defined as CD4^+^ cells (E), and their subpopulations were categorised as naïve (N; CCR7^+^, CD45RO^-^), central memory (CM; CCR7^+^, CD45RO^+^), effector memory (EM; CCR7^-^, CD45RO^+^), and terminally differentiated (TD; CCR7^-^, CD45RO^-^; F). Regulatory T-cells were defined as CD3^+^, CD4^+^, CD25^high^, CD127^-^ cells (G). CD8^+^ T-cells were gated within T-cells (H), and their subpopulations were categorised as naïve (N; CCR7^+^,CD45RO^-^), central memory (CM; CCR7^+^,CD45RO^+^), effector memory (EM; CCR7^-^,CD45RO^+^), and terminally differentiated (TD; CCR7^-^,CD45RO^-^; I).


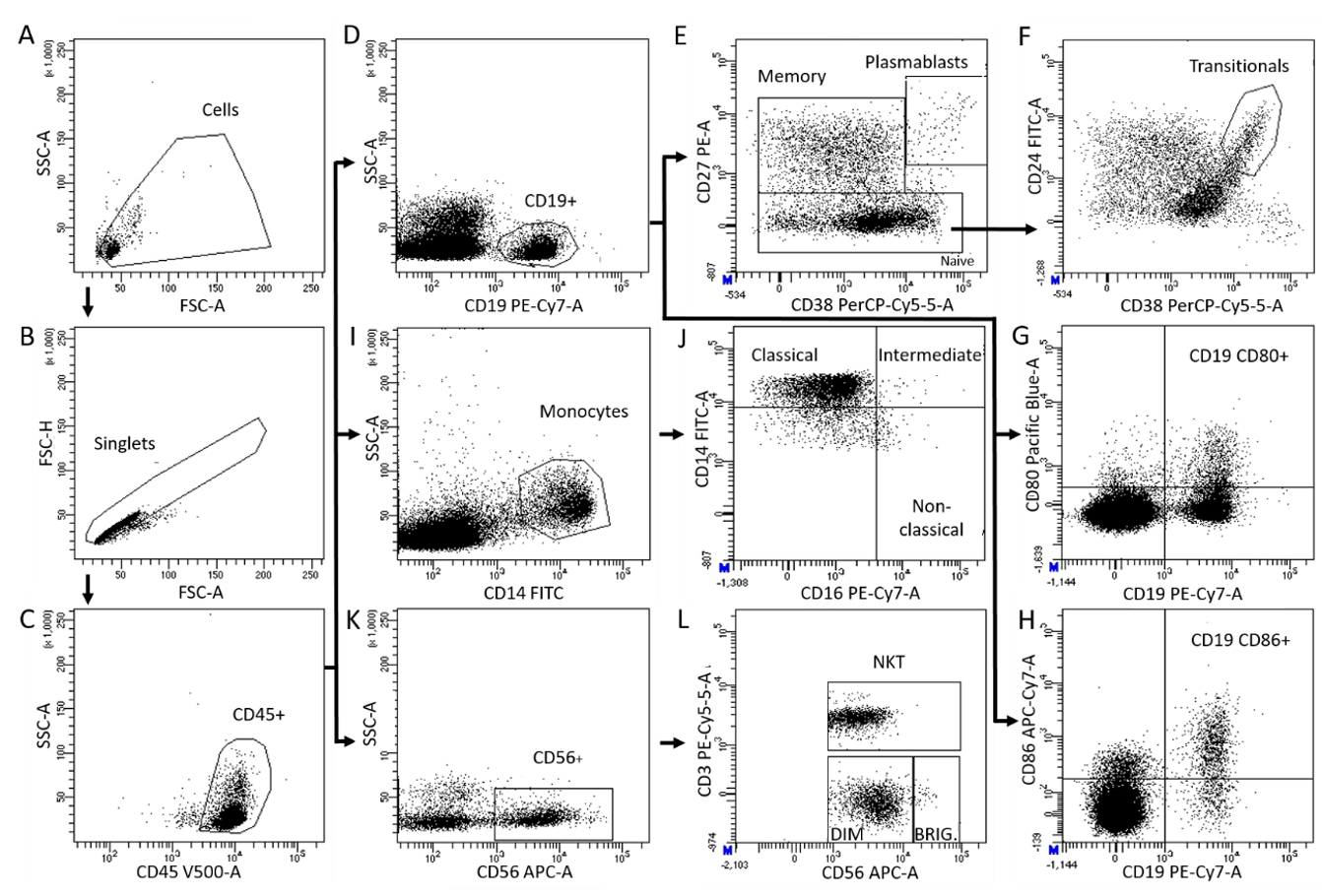


Supplementary Figure 2. Gating strategy for the identification of monocytes, B-cells and CD56^+^ cells. Total events were first gated to exclude apoptotic cells and debris (A) and then gated for doublet discrimination (B). The peripheral blood mononuclear cells (PBMCs) were CD45^+^ (C). B-cells were identified as CD19^+^ (D). B-cells were divided into four subsets: naïve B-cells (CD19^+^, CD38^-^, and CD27^-^), memory B-cells (CD19^+^, CD27^dim^, and CD38^dim^), plasmablasts (CD19^+^, CD27^high^, and CD38^high^; E), and transitional B-cells (CD19^+^, CD27^-^, and CD24^high^, and CD38^high^; F). Antigen-presenting B-cells were identified as CD80^+^ (G) and CD86^+^ (H) cells. Monocytes were defined as CD14^+^ cells (I). Within the monocyte subset, the expression of CD14 and CD16 was used to gate classic (CD14^high^, CD16^⁻/low^), intermediate (CD14^high^, CD16^high^), and nonclassic (CD14⁺, CD16^high^) populations (J). CD56^+^ cells were gated (K), and according to the expression of CD3 and CD56, natural killer T-cells (NKTs; CD56^+^ and CD3^+^); NK CD56dim cells (CD3^-^ and CD56^+/low^); and NK CD56^bright^ cells (CD3^-^ and
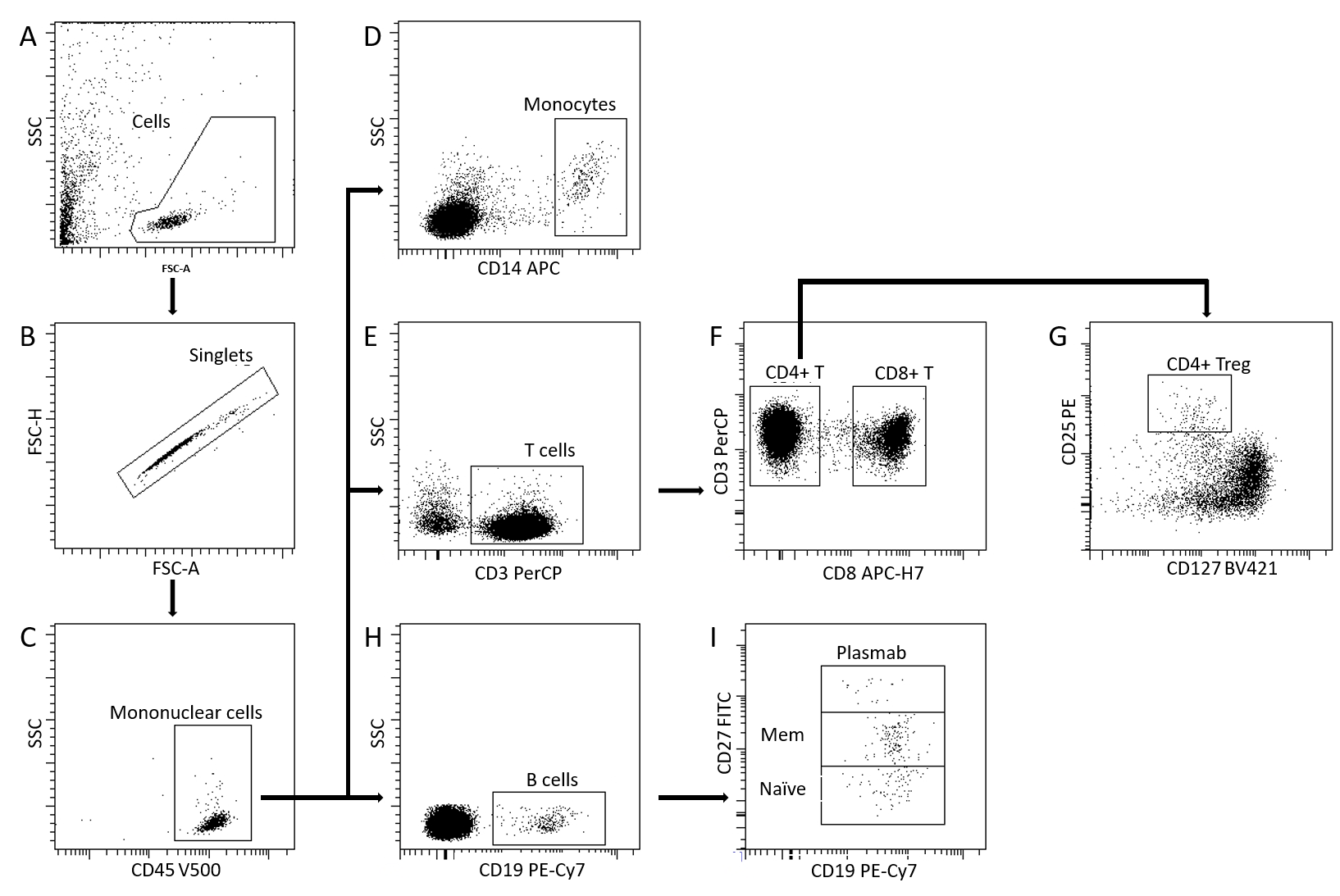
CD56^high^) were identified (L).

Supplementary Figure 3. Gating strategy employed for the identification of CSF leukocyte subsets by flow cytometry. An initial region was set on the forward-area (FSC-A) vs. side-area (SSC) scatter dot plot to exclude debris or apoptotic cells (A), and then a second region was drawn on the FSC-A vs. FSC-height (FSC-H) scatter plot to exclude duplets (B). A third region was set around cells expressing intermediate-to-high CD45 with low-to-intermediate side scatter to select total mononuclear cells (C). Total monocytes were identified as cells expressing high levels of CD14 with intermediate SSC (D). T-cells (E) were selected on the basis of their CD3 expression and classified as CD4^+^ T-cells (CD3^+^ CD8^-^) or CD8^+^ T-cells (CD3^+^ CD8^+^, F). CD4^+^ regulatory T-cells were gated as CD25^high^ cells with CD127^-^ expression (G). B lymphocytes were identified as CD19^+^ cells (H) and subdivided by their CD27 (I) expression into naïve B-cells (CD27^-^), memory B-cells (CD27^+^), and plasmablasts (CD27^++^).

Supplementary table 1. Percentage of cytokine-producing blood immune cells.

| Cell population | HC (n=84) | NLGL (n=45) | NH (n=53) | NLGH (n=19) |
| --- | --- | --- | --- | --- |
| T CD4^+^ TNF-α^+^ | 6.6 [3.5-16.4] | **18.8 [13.2-24.8] ⱡ****** | **18.7 [12.4-26.3] ⱡ****** | **20.6 [14.6-25.9] ⱡ**** |
| T CD4^+^ IL-17^+^ | 0.04 [0.02-0.2] | **0.3 [0.2-0.5] ⱡ****** | **0.2 [0.1-0.4] ⱡ***** | **0.2 [0.1-0.4] ⱡ***** |
| T CD4^+^ IFN-ϒ^+^ | 1.9 [1-4.9] | **4.2 [2.9-8] ⱡ***** | **4.8 [2.9-7.3] ⱡ***** | **5.4 [3.8-7.4] ⱡ*** |
| T CD4^+^ GM-CSF^+^ | 0.9 [0.5-1.9] | 1.3 [0.9-2.1] | **1.5 [0.9-2.5] ⱡ**** | 1 [0.8-2.4] |
| T CD8^+^ TNF-α^+^ | 1.7 [0.8-4.2] | **4.2 [3.2-7.4] ⱡ****** | **4.4 [3.5-5.9] ⱡ****** | **3.9 [3.2-6] ⱡ*** |
| T CD8^+^ IL-17^+^ | 0.02 [0.01-0.1] | **0.14 [0.1-0.2] ⱡ**** | **0.13 [0.05-0.2] ⱡ****** | **0.14 [0.08-0.23] ⱡ**** |
| T CD8^+^ IFN-ϒ^+^ | 1.1 [0.6-3.3] | **2.8 [2.1-4.3] ⱡ***** | **3.0 [2.3-4.6]ⱡ***** | **3.1 [2.4-3.9] ⱡ*** |
| T CD8^+^ GM-CSF^+^ | 0.2 [0.1-0.5] | 0.4 [0.2-0.7] | **0.6 [0.3-1.1] ⱡ****** | 0.3 [0.2-0.6] |
| CD19^+^ TNF-α^+^ | 0.2 [0.1-0.5] | **0.8 [0.3-1.1] ⱡ***** | **0.6 [0.3-1.2] ⱡ***** | **0.9 [0.6-1.3] ⱡ**** |

The values are shown as medians [25-75% IQR]. All cell percentages are shown relative to CD45⁺ cells. Significant comparisons are highlighted in bold. Abbreviations: GM-CSF: granulocyte‒macrophage colony‒stimulating factor; HCs: healthy controls; IFN-γ: interferon gamma; IL-17: interleukin 17; IQR: interquartile range; NH: patients with high sNfL Z-scores; NLGH: patients with low sNfL Z-scores and high GFAP levels; NLGL: patients with low sNfL Z-scores and GFAP values; TNF-α: tumour necrosis factor alpha. ⱡ: significant difference from the HC group *p<0.05, **p<0.01, *** p<0.001, and **** p<0.0001 according to Dunn’s test. In all cases, significant p values in Dunn’s test implied p values < 0.05 in the associated Kruskal‒Wallis test.

Supplementary Table 2. Percentage of NK CD56^dim^ receptors in MS patients and HC.

| Cell population subsets | HC (n=84) | NLGL (n=45) | NH (n=53) | NLGH (n=19) |
| --- | --- | --- | --- | --- |
| Activators |  |  |  |  |
| CD56^dim^ NKG2D^+^ | 93.5 [91.7-94.7] | 94.8 [94.0-95.0] | 94.5 [92.8-94.9] | 94.8 [93.8-94.9] |
| CD56^dim^ NKG2C^+^ | 15.2 [10.8-18.4] | 12.5 [11.8-15.6] | 15.5 [13.0-18.8] | 13.9 [12.2-19.9] |
| Regulators |  |  |  |  |
| CD56^dim^ CD158a+ | 34.3 [29.2-39.8] | 34.5 [28.8-39.2] | 32.8 [30.7-36.3] | 32.2 [29.4-36.7] |
| CD56^dim^ CD158b^+^ | 25.2 [23.8-26] | 20.1 [17.4-27.1] | 23.1 [18.8-30.7] | 24.1 [19.1-31.8] |
| CD56^dim^ CD158e^+^ | 28.8 [26.7-31.3] | 27.7 [23.5-31.6] | 26.5 [23.6-31.6] | 26.3 [23.3-32.7] |
| CD56^dim^ NKG2A^+^ | 12.4 [11.9-14.4] | **19.2 [14.4-26.8] ⱡ *** # ***** | 12.7 [5.9-21.3] | 19.8 [10.8-30.6] |

Values are expressed in median percentage [25%-75% IQR]. All percentages are represented on CD56^dim^ Natural Killer cells. Significant comparisons are highlighted in bold. Abbreviations: HC: Healthy Controls; IQR: Interquartile Range; NH: Patients with high sNfL Z-score; NLGH Patients with normal sNfL Z-score values and high GFAP levels; NLGL: Patients with normal sNfL Z-score and GFAP values group. ⱡ: significant comparison vs HC group; #: significant comparison with NH group; *p<0.05, **p<0.01, ***p<0.001, and ****p<0.0001 Dunn’s test. In all cases significant p values in Dunn’s test implied p-values < 0.05 in the associated Kruskal-Wallis test.

Supplementary Table 3. Percentages of peripheral mononuclear blood cell subsets and intracellular cytokine production by T and B cells present in NH patients showing low (NHGL) and high (NHGH) sGFAP levels.

| Cell population | NHGL (n=19) | NHGH (n=34) | p-value |
| --- | --- | --- | --- |
| Lymphocytes | 84.5 [79.4-80.0] | 85.0 [80-88.9] | 0.95 |
| CD4^+^ T cells | 36.2 [28.0-42.4] | 35.4 [28.4-42.9] | 0.86 |
| Naïve | 10.4 [4.9-15.0] | 9.8 [4.9-15.7] | 0.8 |
| Central Memory | 7.2 [2.7-12.8] | 7.3 [3.6-11.9] | 0.62 |
| Terminally differentiated | 2.4 [1.6-4.3] | 2.9 [1.1-5.7] | 0.90 |
| Effector Memory | 10.15 [5.1-20.9] | 12.6 [5.8-16.0] | 0.91 |
| Regulatory | 0.6 [0.5-1.3] | 0.6 [0.4-0.9] |  |
| CD8^+^ T cells | 20.3 [15.7-24.1] | 19.07 [15.8-26.0] | 0.82 |
| Naïve | 4.8 [2.2-8.1] | 4.8 [3.1-7.5] | 0.88 |
| Central Memory | 0.5 [0.4-1.3] | 0.9 [0.6-1.4] | 0.25 |
| Terminally differentiated | 4.0 [256-9.0] | 4.6 [2.5-8.4] | 0.82 |
| Effector Memory | 6.4 [4.2-9.1] | 6.8 [5.2-12.1] | 0.32 |
| CD19^+^ cells | 6.0 [4.8-8.2] | 6.7 [5.0-9.4] | 0.31 |
| Naive | 3.3 [2.2-4.1] | 3.6 [2.6-5.9] | 0.25 |
| Memory | 2.3 [1.7-3.0] | 2.4 [1.9-3.8] | 0.47 |
| Plasmablasts | 0.07 [0.04-0.12] | 0.06 [0.03-0.1] | 0.61 |
| Transitional | 0.15 [0.08-0.3] | 0.1 [0.05-0.3] | 0.53 |
| CD80^+^ | 1.0 [0.8-1.7] | 1.4 [0.9-1.9] | 0.11 |
| CD86^+^ | 3.5 [1.7-5.0] | 2.8 [1.4-4.5] | 0.54 |
| NK CD56^dim^ | 14.8 [12.1-19.6] | 14.6 [11.2-20.4] | 0.68 |
| NK CD56^dim^ CD122^+^ | 12.9 [9.0-18.9] | 12.3 [9.9-18.4] | 0.71 |
| NK CD56^bright^ | 0.7 [0.4-1.2] | 0.6 [0.4-1.0] | 0.3 |
| NKT | 3.5 [2.0-4.6] | 2.8 [1.5-5.4] | 0.99 |
| Monocytes | 10.7 [6.8-14.7] | 13.8 [7.0-19.4] | 0.11 |
| PD-L1^+^ | 0.15 [0.05-0.4] | 0.2 [0.1-0.3] | 0.49 |
| T CD4^+^ TNF-α^+^ | 20.0 [8.4-28.3] | 17.4 [13.1-21.8] | 0.95 |
| T CD4^+^ IL-17^+^ | 0.3 [0.2-0.4] | 0.2 [0.1-0.3] | 0.38 |
| T CD4^+^ IFN-ϒ^+^ | 4.9 [1.9-7.6] | 4.5 [3.3-6.7] | 0.90 |
| T CD4^+^ GM-CSF^+^ | 1.6 [0.7-2.8] | 1.4 [1.0-2.6] | 0.81 |
| T CD8^+^ TNF-α^+^ | 4.0 [2.8-5.3] | 4.8 [3.7-5.9] | 0.22 |
| T CD8^+^ IL-17^+^ | 0.2 [0.1-0.3] | 0.1 [0.06-0.18] | 0.20 |
| T CD8^+^ IFN-ϒ^+^ | 2.9 [1.8-4.4] | 2.9 [2.3-4.3] | 0.89 |
| T CD8^+^ GM-CSF^+^ | 0.7 [0.3-1.8] | 0.6 [0.7-1.0] | 0.20 |
| CD19^+^ TNF-α^+^ | 0.5 [0.3-0.9] | 0.6 [0.3-0.9] | 0.58 |

The percentage values are expressed as medians [25–75% IQR]. All cell subset percentages were calculated within CD45⁺ cells. Cytokine expression (GM-CSF, TNF-α, IFN-γ, and IL-17) was assessed in CD4⁺, CD8⁺ T cells, and B cells. Values represented the percentage of cytokine-producing cells within each subset. NHGH: patients with high sNfL Z-scores and high sGFAP levels; NHGL: patients with high sNfL Z-scores and low GFAP levels; NKs: natural killer cells; PD-L1: programmed death-ligand 1; GM-CSF: granulocyte-macrophage colony-stimulating factor; TNF-α: tumor necrosis factor alpha; IFN-γ: interferon gamma; IL-17: interleukin-17; IQR: interquartile range. Significant differences were calculated via Mann-Whitney test.

Supplementary Table 4. Percentages of cerebrospinal fluid cell subsets.

| Cell population subsets | NLGL (n=30) | NH (n=44) | NLGH (n=8) |
| --- | --- | --- | --- |
| CD4^+^ T cells | 68.66 (62.78-73.58) | 66.15 (60.19-71.10) | 66.54 (60.32-72.04) |
| Regulatory T cells | 2.81 (2.04-3.9) | **1.74 (1.2-3.0)** ¶****** | 2.24 (1.61-2.68) |
| CD8^+^ T cells | 18.95 (16-21.86) | 19.4 (16.05-24.11) | 18.72 (15.75-21.42) |
| CD19^+^ cells | 2.81 (2.31-4.14) | 2.61 (1.81-4.85) | 2.42 (1.42-4.12) |
| Memory B cells | 1.88 (1.38-2.82) | 1.7 (1.2-2.79) | 1.84 (1.25-2.71) |
| Monocytes | 2.25 (1.37-3.84) | 2.05 (0.99-3.99) | 2.12 (1.07-3.78) |

The values are shown as the median percentages [25-75% IQR]. All percentages are expressed as proportions of CD45⁺ cells. Significant comparisons are highlighted in bold. Abbreviations: IQR: interquartile range; NH: patients with high sNfL Z-scores; NLGH: patients with low sNfL Z-scores and high GFAP levels; NLGL: patients with low sNfL Z-scores and GFAP values. ¶: significant difference compared with the NLGL group; **p<0.01 according to Dunn’s test. A significant p value in Dunn’s test implied a p-value < 0.05 in the associated Kruskal‒Wallis test.
